# Citizen science is a vital partnership for invasive alien species management and research

**DOI:** 10.1016/j.isci.2023.108623

**Published:** 2023-12-03

**Authors:** Michael J.O. Pocock, Tim Adriaens, Sandro Bertolino, René Eschen, Franz Essl, Philip E. Hulme, Jonathan M. Jeschke, Helen E. Roy, Heliana Teixeira, Maarten de Groot

**Affiliations:** 1 UK Centre for Ecology & Hydrology, Wallingford, Oxfordshire, UK; 2 Research Institute for Nature and Forest (INBO), Brussels, Belgium; 3 Department of Life Sciences and Systems Biology, University of Turin, Turin, Italy; 4 CABI, Delémont, Switzerland; 5 Division of BioInvasions, Global Change & Macroecology, Department of Botany and Biodiversity Research, University of Vienna, Vienna, Austria; 6 Bioprotection Aotearoa, Department of Pest Management and Conservation, Lincoln University, PO Box 84850, Christchurch, Lincoln 7648, New Zealand; 7 Leibniz Institute of Freshwater Ecology and Inland Fisheries (IGB), Berlin, Germany; 8 Institute of Biology, Freie Universität Berlin, Berlin, Germany; 9 Centre for Environmental and Marine Studies, Department of Biology, University of Aveiro, Campus de Santiago, Aveiro, Portugal; 10 Slovenian Forestry Institute, Večna pot 2, 1000 Ljubljana, Slovenia; 11 Centre for Ecology and Conservation, Faculty of Environment, Science and Economy, University of Exeter, Penryn, United Kingdom

**Keywords:** Natural sciences, Nature conservation, Ecology

## Abstract

Invasive alien species (IAS) adversely impact biodiversity, ecosystem functions, and socio-economics. Citizen science can be an effective tool for IAS surveillance, management, and research, providing large datasets over wide spatial extents and long time periods, with public participants generating knowledge that supports action. We demonstrate how citizen science has contributed knowledge across the biological invasion process, especially for early detection and distribution mapping. However, we recommend that citizen science could be used more for assessing impacts and evaluating the success of IAS management. Citizen science does have limitations, and we explore solutions to two key challenges: ensuring data accuracy and dealing with uneven spatial coverage of potential recorders (which limits the dataset’s “fit for purpose”). Greater co-development of citizen science with public stakeholders will help us better realize its potential across the biological invasion process and across ecosystems globally while meeting the needs of participants, local communities, scientists, and decision-makers.

## Graphical abstract

**Figure:**
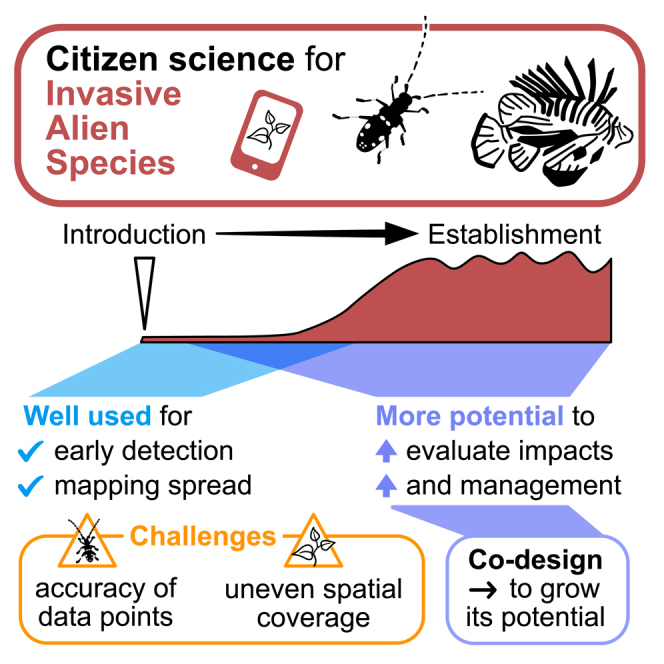

## Introduction

Invasive alien species (IAS) are plants, fungi, animals, and microbes that have been transported via human agency to a location beyond their natural range. What marks them out from other alien, non-native species is the negative impacts that they have on native biodiversity, ecosystem functions, and human livelihoods, health, and well-being.^1^^,^^2^^,^^3^ Indeed, IAS are important drivers of native biodiversity loss,^2^^,^^3^ and the economic costs of their impacts are substantial and increasing.^4^ This means that negative impacts of IAS need to be managed.^5^^,^^6^ Effective management of IAS and their impacts is required under the Kunming-Montreal Global Biodiversity Framework (Target 6),^7^ but ultimately this depends on having adequate information, gained through the collection and analysis of data.^8^

The breadth of IAS impacts means that they are often of direct concern to the public.^9^ The public can contribute to knowledge, and hence action, through the collection of data in “citizen science” or “community science” initiatives. (Here, we use the widely used term “citizen science” to encompass the diversity of the ways in which public audiences voluntarily participate in scientific research and monitoring.) This typically includes data collection in the field^10^ but can include sample collection for later analysis,^11^ project design, or using results for management.^12^ Citizen science can be a cost-efficient approach for surveillance and research at large spatial extents,^13^ and so the data are a crucial source of evidence informing IAS policy, decision-making, and management across the world^3^^,^^10^^,^^14^^,^^15^^,^^16^^,^^17^*.* Public participation through citizen science has an important role in awareness raising,^3^ creating learning opportunities, and enabling civic engagement^18^^,^^19^ as well as in policy development^20^ and environmental management for IAS,^14^^,^^21^ all of which can contribute to reducing the establishment, spread, and impacts of IAS.

Citizen science has been applied to IAS across terrestrial,^22^^,^^23^ freshwater,^24^ and marine^25^ ecosystems. To date, however, much of the focus of IAS citizen science (including the focus of policy) has been on early detection of IAS and surveillance of spread^2^^,^^10^^,^^26^^,^^27^ (see the section “Citizen science can be used across the biological invasion process”). Overall this means that, currently, citizen science is not strategically used to its full potential for IAS surveillance, monitoring, and research.

Here we review the opportunities and challenges of using citizen science to meet knowledge needs for IAS across the stages of the biological invasion process. We highlight the diversity of citizen science approaches, how they can be effective at different stages in the invasion process, and how they could be used more. We also explore two important challenges for the use of citizen science for IAS: (i) ensuring data quality, especially reducing misidentification of taxa, and (ii) dealing with uneven spatial coverage of recording effort.

### Citizen science can be used across the biological invasion process

**Figure 1:**
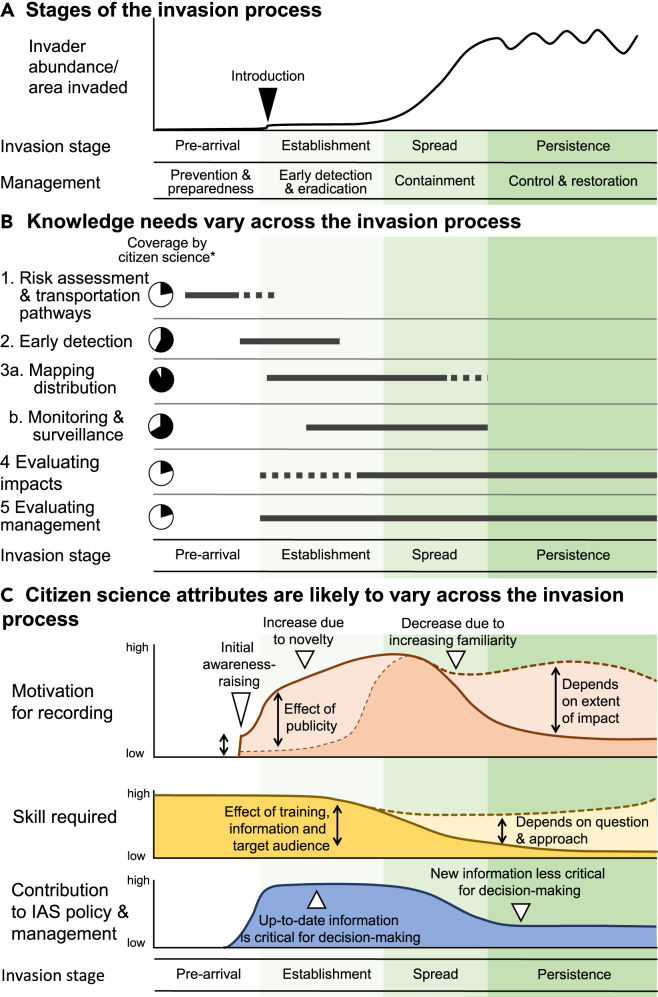
How citizen science varies across the biological invasion process (A) The stages of the process of invasive alien species (IAS) spread and establishment and the consequent management requirements (based on Roy et al.^3^). (B) Different types of information are required across the stages of the biological invasion process. (C) The attributes of citizen science are likely to vary across the stages of the biological invasion process, as illustrated here based on the authors’ experience. Specifically we consider participant motivation, required skills, and the contribution to IAS policy and management. The stages of the invasion process are shaded across sections a, b, and c. ∗Coverage by citizen science shows (in black) the proportion of projects addressing each knowledge need from 103 IAS citizen science projects from Europe.^10^ See Table S1 for full details.

The biological invasion process can be described as a series of sequential stages from arrival to persistence (Figure 1A).^3^^,^^28^ The management responses, and thus the information needed for decision-making, vary across these stages (Figure 1B and Table S1).^10^ However, the focus of citizen science is mostly in the establishment and spread phases of the biological invasion process and to support public engagement^3^ (Figure 1B).

For citizen science to be “successful” (which here we define as being fit for purpose to address the questions of interest), it needs an interdisciplinary approach, drawing on the expertise of social science as well as natural science researchers.^29^ This is because successful IAS citizen science relies on applying ecological knowledge about the species but also relies on (i) people’s motivation to participate (a “bottom-up” driver^30^), (ii) the skills required for participation, and (iii) the need for information (e.g., in policy decision-making or by local people: a “top-down” driver). Our experience suggests that the balance of these three social components will vary across the biological invasion process, as proposed in Figure 1C. For instance, early detection is vital for rapid response by authorities responsible for IAS management. At this stage, people’s awareness of the problem could be high due to awareness-raising campaigns, especially if the species is charismatic^31^^,^^32^ or viewed as a threat,^33^ although resources are needed to boost people’s skills in identification. For instance, the “Check a Tree” month promoted by the US Department of Agriculture in August 2023 was designed to raise awareness of Asian longhorn beetle *Anoplophora glabripennis*, as well as promote citizen science reporting.^34^ In contrast, once an IAS has become established, people may have already encountered the species, so the imperative for reporting will be different—people might be less inclined to report each sighting but could be motivated to report in response to management^6^ or negative impacts of the IAS.^35^ Therefore, different citizen science approaches are likely to be most useful at different stages.

**Figure 2:**
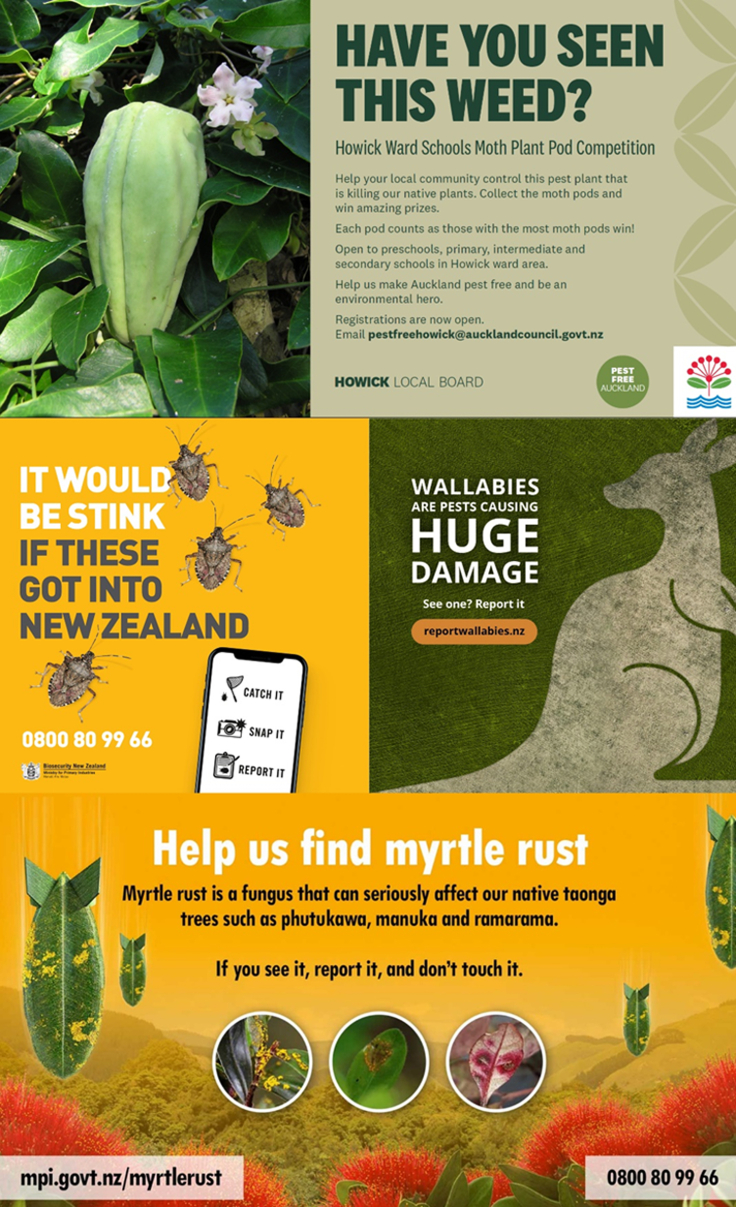
Examples of resources produced by national and local government agencies in New Zealand to raise awareness and encourage the reporting of an invasive alien weed (moth plant *Araujia sericifera*), invertebrate (brown marmorated stinkbug *Halyomorpha halys*), and vertebrate (Bennetts wallaby *Macropus rufogriseus*), and a fungal pathogen (myrtle rust *Austropuccinia psidii*) Note the gamified, competitive approach used to encourage school children to report moth plant *Araujia sericifera*.

Throughout the stages of the biological invasion process, publicity and awareness-raising can influence the success of citizen science (Figure 1C). Awareness raising has been a key action for government agencies in New Zealand,^16^ and it appears to be effective because 35%–40% of the New Zealand population state that they “always” or “usually” keep an eye out for unusual pests or weeds—potentially an additional 1.5 million pairs of eyes for citizen-based biosecurity surveillance.^36^ This includes citizen science reporting of species that (i) pose a new risk to New Zealand if they arrived (e.g., brown marmorated stinkbug *Halyomorpha halys*), (ii) are the focus of an eradication campaign (e.g., myrtle rust *Austropuccinia psidii*), or (iii) might be spreading out from a containment zone (e.g., Bennett’s wallaby *Macropus rufogriseus*) (Figure 2).

### The diversity of citizen science approaches and their use for IAS

It is important to understand that citizen science is not a single approach. Different citizen science approaches will likely vary in their usefulness in the different stages of the biological invasion process. Firstly, citizen science projects vary in their methodology^37^^,^^38^^,^^39^^,^^40^: from unstructured activities (including “opportunistic” recording in which people make records where and when they choose) through to more structured, scientific sampling (e.g., repeatedly following a protocol in set locations) and from simple projects (e.g., simply submitting a photo) to elaborate projects more akin to mini research projects^40^ (Table 1). Secondly, citizen science projects vary in their audiences. The target audience of many projects is the general public,^10^ but it may be more effective to target specific audiences, such as recreational divers,^41^ bee-keepers,^42^ or hunters,^43^ based on their likelihood of encountering an IAS and their motivation to participate in citizen science. Thirdly, projects can be begun by different people: many citizen science activities are initiated by professional scientists or authorities, but they could be initiated by local communities concerned by the potential impact of IAS or who want to test management interventions. Participatory monitoring, initiated by local communities, also occurs in other fields of environmental monitoring and provides a model for how this can be developed.^44^ Activities can be focused on the IAS as the target, begun by authorities raising awareness of biosecurity (e.g., with the public simply reporting things that are “out of the ordinary”) or begun by organizations interested in wildlife recording (with IAS data being a byproduct).

**Table 1:** A simple typology of citizen science approaches, their characteristics, limitations, and benefits

Citizen science approach^a^	Characteristics	Benefits	Limitations	Example for IAS
Simple and unstructured	Recording from wherever and whenever the participant chooses. Data are typically limited, e.g., location and species identity, with corroborating evidence such as a photograph	• Opportunity for mass participation and gathering many records • Only requires one-off contributions • Can engage people with little experience	• Datasets can be challenging to analyze rigorously	• Early detection of invasive pests and diseases^45^ • Adding value to routine monitoring in coastal areas^46^
Structured	Following a protocol at selected places (sometimes chosen by the project organizers), often requiring repeated visits to the site	• Produces high-quality datasets because recorder “effort” has been controlled • Can be designed for representative coverage of a region • Records from repeated visits provide high-quality data on change	• Requires higher level of commitment, so participation is typically lower than unstructured • Therefore requires higher investment in volunteer retention and training	• Sentinel trees for monitoring invasive pests and diseases^45^ • Effect of the emerald ash borer on woodpecker populations^47^ • Assessing changing abundance of invasive plants^48^ • Monitoring vertebrate IAS management^49^
Elaborate	More complex activities to gather data following a detailed protocol or experimental design	• Can provide rich, contextual datasets for quantifying impacts or drivers of change	• Requires higher level of commitment, so participation is typically lower than unstructured	• Attack rate of parasitoids on *Cameraria ohridella*^50^ • Local ecological knowledge of fishers to map IAS distribution and identify dispersal vectors^51^

Typology as adapted from Pocock et al.^40^

a This categorization is a simplification, and there are many projects that fit between these categories, e.g., semistructured citizen science in which people can take part where they choose but follow a protocol (e.g., searching for a fixed length of time) so that records are comparable with each other.^39^

## Opportunities for citizen science across the biological invasion process

How can citizen science be useful across the stages of biological invasion,^28^ namely pre-arrival; introduction and establishment; spread; and persistence, so as to provide data to support prevention, early detection, containment, control, and ecological restoration (Figure 1)? Here, we do not provide a comprehensive review of all projects but instead provide illustrative examples to explore how citizen science is used and could be used better. The key opportunities, as discussed in the following, are summarized in Table 2.

**Table 2:** Summary of key challenges for IAS citizen science as discussed in the text

Knowledge needs	Stage of the biological invasion process^a^	Key opportunities for citizen science as discussed in the text
Risk assessment and pathways of introduction	Pre-arrival	• Collate data on non-naturalized species (“casual” plants and “escapes”) and presence of cultivated/domesticated species to inform risk assessments • Gain better data on native species, including species interactions, to inform ecological impact assessment of IAS • Sentinel monitoring to identify species with potential to become invasive elsewhere
Early detection	Introduction and establishment	• Gain better ways of estimating recorder coverage • Ensure rapid data flow for positive records • Monitor the success of rapid response efforts to eradicate new IAS after early detection • Develop and test training and technological tools to support accurate identifications (see the section “ Challenge 1: Accuracy of data points, especially reducing misidentifications”)
Monitoring distribution spread	Spread	• Ensure rapid data flow for positive records, especially showing range expansion • Use structured and semistructured approaches more to gain rigorous systematic information on absences/non-detections • Use methods to avoid bias in results due to uneven spatial coverage of recorders (see the section “Challenge 2: Dealing with uneven spatial coverage”)
Assessing impacts	Spread, Persistence	• Explore how citizen science can be used more to assess the impacts of IAS • Gain data on species abundance via standardized protocols • Collect standardized data on ecological and socio-economic impacts
Evaluating management success	Establishment, Spread, Persistence	• Explore how citizen science can be used more to support adaptive management of IAS • Use structured and elaborate citizen science with experimental designs (before-after, control-intervention) • Involve volunteers in management actions as well as data collection • Record occurrence of management actions

a The stage for which the knowledge needs are most relevant, based on Figure 1B. This is indicative rather than exhaustive.

### Risk assessment and pathways of introduction

#### Citizen science data can inform horizon scanning and risk assessment

Before a species has first become introduced into a region beyond its natural range, citizen science can contribute biological information in its current (native and/or invaded) range. This helps to inform “horizon scanning” activities, which is a risk assessment used to target attention on the IAS of greatest concern.^52^^,^^53^ Global sources of distribution data, such as the Global Biodiversity Information Facility (GBIF), include many citizen science species records.^10^^,^^54^ Analysis of these data provides insights about habitat preferences and bioclimatic limits, which can be used in horizon scanning to predict areas of future establishment.^55^

One part of horizon scanning is to quantify the likelihood of arrival, release, or escape of alien pests into the environment, and citizen science can help with this. For instance, the Plant Alert project in the UK surveyed the occurrence of alien plants in gardens and their mode of natural reproduction^56^^,^^57^ to identify which species might “jump the garden fence” and become invasive.^16^ Open publication of data on “casual”, non-native plant species and non-naturalized escapee animals would help inform these risk assessments.^58^ The Darwin Core standard (a way of standardizing data on biodiversity records) has recently been extended to include terms such as “degree of establishment” and “pathway” (of introduction) to make IAS records easier to use and share.^59^ As well as escaping from captivity/cultivation, trade pathways are another route of arrival. Monitoring trade pathways is usually undertaken by professionals, although public information such as that offered through legal or illegal trade sites on the internet could help to inform risk of arrival.^60^^,^^61^ Citizen science could be useful to monitor specific trade pathways, but we are not aware of this having been developed; it could raise substantial issues with reporting biases and ethical or regulatory risks.^62^

Impact assessment is another important element of horizon scanning. Citizen science data on native species, such as data on habitat, phenological overlap with a potential IAS, or species interactions, can help inform scientists about potential impacts of IAS on native biodiversity. For example, niche overlap indices with native species informed the ecological impact assessment for an invasive alien ladybird *Harmonia axyridis*.^63^ Volunteer recorders may not be aware of the secondary uses of their data for these purposes, so providing feedback to them might stimulate more recording, especially of species interactions,^64^^,^^65^ thereby increasing knowledge on the ecology and potential impacts of IAS.

#### Sentinel monitoring for future invasive species

“Sentinel” monitoring is when key sites are monitored for potential IAS, for example, reporting pests and diseases on non-native trees in arboreta or botanic gardens to detect species that could become invasive.^66^ Currently, sentinel tree monitoring is undertaken mostly by professionals, but this is costly and it could be undertaken through citizen science. Trained volunteers could use protocols at set locations to identify potential-risk species to augment data from professionals.^67^

### Early detection of IAS

#### Early detection is vital to reduce establishment of IAS

Preventing the arrival of IAS is the most effective way to address the threat of biological invasions.^2^ However, despite implementation of biosecurity approaches, including pathway management and border biosecurity, IAS incursions are increasing,^68^ so it is essential to have an early warning system to detect new species before their establishment and spread. Mass participation through “unstructured” citizen science plays an internationally recognized role in early detection,^3^ and it would be prohibitively expensive to achieve such high coverage with contracted staff.^69^^,^^70^ Important challenges for early detection with citizen science are gaining sufficient spatial coverage for effective early detection, efficient confirmation of putative detections, and rapid data flow to responsible authorities.

With more people informed and engaged with early detection, the chance of rapidly detecting IAS incursions is increased, but it is hard to identify false absences, i.e., the lack of observers from the absence of the IAS, without more structured information (Table 1). The spatial coverage of potential recorders is uneven,^71^ but if detection rate and spatial coverage are known, the probability of early detection can be empirically estimated and used to design monitoring strategies, such as targeted professional surveillance to fill gaps in coverage.^72^ Because the issue of uneven coverage is so important, possible solutions are discussed further in the section “Challenge 2: Dealing with uneven spatial coverage.”

#### Mass participation citizen science can support early detection of IAS

There are several species-recording platforms for citizen science (e.g., iNaturalist and Observation.org), and these can provide useful data on early detections, including countries in the Global South that tend to have less citizen science recording.^73^ The number of potential IAS is large, so the effectiveness of mass participation citizen science can be boosted by selecting focal species to target, such as informed by horizon scanning^52^ (Figure 2 and Table 2). For example, the Asian Hornet Watch app was developed in the UK for reporting *Vespa velutina* (https://www.brc.ac.uk/app/asian-hornet-watch) prior to its arrival in the UK and was promoted to both the general public and bee-keepers (a community with a high chance of making early detections). It includes a comprehensive identification guide, and submission via the app ensures rapid data flow to verifiers and relevant authorities.

Valuable data on early detection of IAS is sometimes circulated on social media forums. One innovative approach that has been trialled is using chatbots on Facebook sites to autonomously interact with recorders and inform them how to submit their records.^74^ The combination of chatbots with automated image recognition would be an innovative approach for the early detection of photogenic species, although lack of knowledge about the spatial coverage of such reporting would remain an issue (see the section “Challenge 2: Dealing with uneven spatial coverage”).

Misidentification is a challenge for early detection partly because the chance of detection by any single individual is low.^75^ While the benefit of making an early detection of an IAS is high, the cost of verification by professionals (where this is needed) can also be high. This can create an ethical issue regarding the use of citizen science.^62^ Options for verifying data are discussed further in the section “Challenge 1: Accuracy of data points, especially reducing misidentifications.”

#### Structured approaches and diagnostic technologies can enhance early detection with citizen science

Mass participation may be great for “passive surveillance” for larger, more conspicuous species,^76^^,^^77^ but it is more challenging to detect less conspicuous species, like microorganisms, fungi, or many aquatic IAS. (Aquatic species are often hard to detect unless near the shore or by specific groups such as fishers or divers.^78^^,^^79^) To increase the range of species suitable for early detection, new diagnostic technologies, such as DNA analysis, can be used. The ease of sample collection makes it easy to participate, and it can be combined with structured monitoring (Table 1) to gain scientifically rigorous data. Examples of this include water sampling to detect novel *Phytophthera*^80^ or invasive crayfish,^81^ sampling ticks (Acari: Ixodidae) for analysis of zoonotic diseases,^82^ or detecting forest tree pests.^83^ Alternatively broad-spectrum biomonitoring (identifying lots of taxa from a single environmental sample) can be provided by metagenomic methods, such as nanopore sequencing.^84^

One of the reasons that structured monitoring is so valuable for “active surveillance” of IAS is that it provides consistent information to support early detection^19^ as well as providing information about detection probabilities (i.e., false-negative rates) and recorder coverage^85^ (Table 1). Furthermore, adaptive sampling approaches^86^ could be deployed by identifying priority locations for recording^87^ to direct the recording effort by volunteers.

If a report of an unwanted IAS is made, regulatory authorities can undertake further risk assessment and on-the-ground surveys. Sometimes this can lead to successful management. For instance, most early detections of Asian longhorn beetle *Anoplophora glabripennis* are made by members of the public and then confirmed by regulatory authorities,^88^ which has led to its successful eradication after arrival in the UK.^89^ Where management occurs in response to early detection, feedback is important to motivate volunteer vigilance, but when providing feedback it should be remembered that some people will oppose IAS management, especially of vertebrates.^90^ Citizen science could also be used to monitor the success of eradication attempts: trained local people could be cost-effective at “keeping an eye” out for re-emergence of the IAS (see also the section “Evaluating the impacts of management”).

### Monitoring expansion

#### Unstructured recording is valuable for monitoring expansion of IAS

Alien species can spread within a region by natural dispersal and anthropogenic pathways. The rate of spread varies between species and may be rapid.^91^ Unstructured, mass participation citizen science has a major role in monitoring IAS expansion (Figure 1B),^10^ and it may be easier to engage people then compared to the introduction stage because the perceived threat is more real and chance of encounter is higher (Figure 1). For instance, mass participation recording has been effective to gain accurate information about the spread of the Asian tiger mosquito *Aedes albopictus* across Spain, providing a vital public health service.^92^ Smartphone apps, in particular, facilitate recording by the public and can provide good feedback for volunteers, e.g., a map of live sightings or other gamified elements.^93^ Mass participation citizen science can also enable data gathering in places that would be otherwise hard to monitor, for instance tracking the spread of an invasive termite in homes in Taiwan.^94^ However, it must be remembered that the distribution of presence-only records will be dependent on the distribution of recording effort (see the section “Challenge 2: Dealing with uneven spatial coverage” for solutions to this challenge).

#### Structured monitoring could be used more to monitor expansion of IAS

We propose that structured monitoring could be used more widely for monitoring IAS spread by following a protocol at fixed sites.^39^ This would provide data to better estimate true absence rates (taking account of imperfect detection).^95^^,^^96^ Monitoring sites can be selected to provide good spatial coverage. Along the coast of the eastern USA, for instance, a network of pre-selected sites was used for long-term citizen science monitoring tracking the distribution of hard-to-identify invasive alien crabs. Indeed, volunteers recorded not only their presence but also sex and reproductive status, providing additional valuable information on likely establishment.^97^ Structured and unstructured monitoring can be combined. For instance, in Germany, a nationwide surveillance program was initiated in 2011 for monitoring mosquitoes through systematically operated traps. This has run alongside the “Mückenatlas” (mosquito atlas), a passive, “unstructured” citizen science project.^98^ Data from both sources could be combined using integrated modeling to estimate spread.^99^

### Assessing the impacts of IAS

#### There is untapped potential of citizen science to assess the impacts of IAS

While alien species can have impacts throughout the biological invasion process, these impacts are often not well-supported with evidence.^3^^,^^100^ Citizen science could have a valuable role in filling this data gap, but we argue that its potential has been under-used thus far. Abundance of an IAS is often typically recorded in citizen science,^101^ so we recommend investing in methods to simply and consistently record IAS abundance as a proxy for its likely impact.^102^ Even coarse-scale impact data are useful, for instance, the broad categories of leaf damage by the leaf-miner *Cameraria ohridella* as recorded in the Conker Tree Science project.^50^

There is untapped potential for using scientific sampling designs in citizen science, such as before-after or space-for-time comparisons, to obtain evidence on the impact of IAS. Data from long-running citizen science initiatives are particularly valuable in assessing before-after assessment of impacts of IAS on biodiversity. For instance, opportunistic, unstructured records were used to assess the impact of harlequin ladybird *H. axyridis* on native ladybird species in Europe,^103^ records from bird-watchers were used to assess the impact of the emerald ash borer beetle *Agrilus planipennis* on hole-nesting birds in North America,^47^ and public surveys provided information on data on diet of invasive rose-ringed parakeets *Psittacula krameria* in urban South Africa.^104^ Sometimes, valuable data on IAS impact is collected for a different purpose. For instance, families in Iceland have collected the down of eider ducks *Somateria mollissima* and monitored the size of breeding populations on their islands for generations. These data have been invaluable for assessing the impact of American mink *Neogale vision* on the eider ducks.^105^

#### Local knowledge can be valuable to assess ecological, social, and economic impacts of IAS

Some communities have “local ecological knowledge” that is not based on survey data. For example, fishers have high-quality knowledge of alien-native species interactions and perceived changes and impacts on local ecosystems.^106^ While care needs to be taken to avoid perception bias when using local ecological knowledge, it could be put to greater use in assessing IAS impacts.^51^

Here we have focused on ecological impacts, but data on socio-economic impacts are even sparser, e.g., Allmert et al. and Evans et al.^107^^,^^108^ These data could be gathered through citizen science monitoring, although questions such as privacy, ethics, and rigorous study design would need to be addressed. One study engaged school children in Florida in DNA analysis of lionfish *Pterois volitans* gut contents to discover its impact on economically important prey, as well as support public engagement with research.^109^ Some information on human impacts of IAS could be obtained via sentiment analysis of publicly available data from social media.^110^

### Evaluating the impacts of management

#### There is untapped potential for citizen science to support adaptive management of IAS

The management of IAS includes local eradication, spatial containment, population control, asset protection, and biocontrol. All these actions require information to evaluate their effectiveness, on changes in both the focal IAS and its impacts. This will support adaptive management strategies that are needed for cost-effective IAS control.^3^

Participatory management of established IAS already involves local organizations or community groups making it ideal for sustained, long-term citizen science.^111^ For instance, a local group working for ecological restoration in Auckland, New Zealand, encourages those undertaking pest control to contribute to both unstructured citizen science recording of IAS and structured recording of trapping success.^49^ However, motivations of these volunteers could change over time. For instance, the motivation of recreational divers to record lionfish *Pterois volitans* in part of the Caribbean declined during the period of its management as its presence became less novel.^112^ Understanding volunteer motivations is, therefore, crucial; depending on the local context, they can include intrinsic concerns about conservation, as well as motivations connected to livelihood and well-being.^9^^,^^111^ We recommend that structured approaches with repeatable methodologies would provide the most rigorous approach for evaluating management actions on IAS and/or its impact, although it will need to be co-designed with volunteers. Nonetheless, unstructured citizen science data remain useful for evaluating management, such as the use of iNaturalist data to track the presence and establishment of a Lepidopteran biocontrol agent of the invasive weed *Chromolaena odorata* in south-east Asia.^113^

#### Citizen science could support monitoring of ecological restoration

Finally, volunteers could be involved in more complex projects that link to ecological restoration. For instance, volunteers have been involved throughout the northeastern USA in locating surviving hemlock trees, *Tsuga canadensis* and *T. caroliniana*, that may be naturally resistant to infestations from the hemipteran hemlock woolly adelgid *Adelges tsugae*^114^ and could be used in plant breeding programs to develop pest-resistant native stock. Linking participation to positive actions for nature could also support people's longer-term motivation for involvement.

## Challenges with the use of citizen science for IAS surveillance, management, and research

Despite the many advantages of citizen science, there are challenges with its use. Citizen science may be free at the point of submission, and the value of data may be vast,^115^ but supporting citizen science is not free: it requires staff to recruit and support volunteers, funding to develop web and app resources, and staff to analyze the results.^13^ Therefore, it is important to ensure that the data are fit for their intended purpose^116^ and that all the dimensions of citizen science data quality—accuracy, relevance, reliability, and completeness—are met.^117^ Here we explore the potential solutions to two dimensions of data quality: accuracy of data points and uneven spatial coverage of recorders.

### Challenge 1: Accuracy of data points, especially reducing misidentifications

A perceived lack of data accuracy is one major reason why citizen science is challenged or mistrusted.^118^^,^^119^^,^^120^ For instance, in New Zealand the general public contributes more than two times the number of reports of IAS to the government agencies compared to all other sources combined,^121^ but people’s confidence in their identification skills is low.^36^ False-positive rate from the general public is substantially higher than other (professional) data providers,^121^ although members of the public could be conservative in reporting any possible sighting. Ways to deliver sufficient levels of accuracy should be considered in the design phase of citizen science projects and addressed through clear protocols, the use of adequate technology, or the provision of training and feedback to volunteers.^96^

Lack of geolocation precision can be problematic with IAS early detection because authorities must respond rapidly when attempting eradication and so require good information about detections. This is less of a problem later in the biological invasion process. Smartphones and map-based website submission have greatly reduced spatial inaccuracy or errors in data transfer and are now ubiquitous in IAS citizen science.^93^

**Figure 3:**
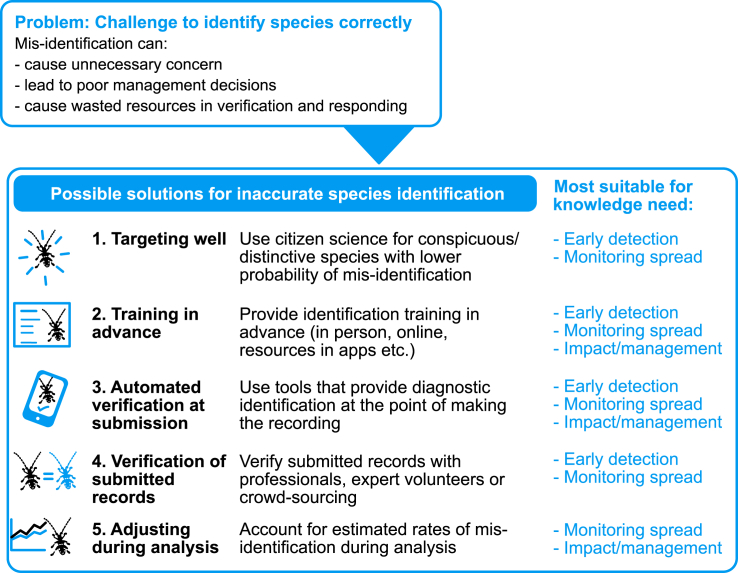
Summarizing the possible solutions for inaccurate species identifications These are discussed in the main text. We have indicated the main knowledge needs addressed by each proposed solution.

In contrast, accurate species identification remains an important challenge, especially in the early stages of the biological invasion process (Figure 1). Misidentification can be costly: reports of new pests require verification by experts, which is time-consuming.^122^ Good, trusted relationships between project organizers and responsible authorities are necessary to ensure rapid, consistent pathways for data flow, verification, and dealing with privacy concerns. Contentious sightings can be hidden from public access, for example, where controversial management actions are required or when records are unconfirmed, although records should be made open as early as possible to ensure their use and effective re-use. Here we consider several options for solutions to the problem of inaccurate species identifications (Figure 3).

#### Targeting well

The likelihood of species reporting rates varies across species,^123^ so it is most fruitful to develop citizen science for species that are easier for members of public to detect and identify. This can still represent a wide diversity of species (e.g., Figure 2) but means that citizen science may not be suitable for some harder-to-identify or harder-to-detect IAS.^124^ Specific audiences can be targeted to meet the required identification skill for target taxa, e.g., recreational fishers instead of the general public. Later in the biological invasion process, IAS are likely to be abundant and/or familiar; thus, misidentification may occur less frequently.

#### Training in advance

Because data verification is time-consuming and costly,^122^ reducing the rate of false positives through training in advance of submission may be cost-efficient.^125^ With appropriate training, volunteer recorders can perform very well at identification, for instance, reporting plant abundance at fixed plots.^126^ Training can be provided in many ways, e.g., through personalized workshops,^45^ online training resources,^125^ or identification guides within smartphone recording apps.^93^

#### Automated verification at submission

Verification can be conducted at the point of submission. Firstly, recorders can be alerted to possible errors with automated outlier detection procedures (e.g., to detect records that appear to be out of geographic range or out of season), and this could be further developed using artificial intelligence or model-based outlier detection.^117^ Secondly, identification can be directly verified using automated image or sound recognition using artificial intelligence, e.g., Hart et al.^127^ Developing this could be cost-efficient to support data quality across the biological invasion process. Thirdly, cheap diagnostic sensors are increasingly likely to be used in citizen science; for example, environmental DNA (eDNA)-based techniques can provide diagnostic identifications for early detection,^60^ e.g., *Phytophthora* in water^80^ or hard-to-detect marine species.^79^ It would be even more useful if eDNA diagnostics were available at the point of use. This is being developed currently with portable PCR (polymerase chain reaction) for forest pests (fungi, oomycetes, and an insect)^83^ or nanopore sequencing^84^ and is likely to become increasingly accessible for use in citizen science in the next few years.

#### Verification of submitted records

Especially in the early stages of the biological invasion process, data will need to be verified after submission to ensure accuracy. This already happens in the vast majority of European citizen science projects, according to Price-Jones et al.^10^ In many cases, data (typically images) submitted by participants are confirmed by experts,^128^^,^^129^^,^^130^ but some projects use peer (community) validation, such as crowdsourcing^131^ or a group of trained volunteers.^132^

#### Adjust during analysis

It is nearly impossible to ensure all data points are accurate, but, if false-positive and false-negative rates are calculated, they can be incorporated in statistical analysis.^122^^,^^133^^,^^134^ Misidentification rates could be estimated in advance through pilot work^50^ or could be estimated directly from repeated sampling data as obtained through structured citizen science.^135^ Errors in individual data points will persist, so this is better for assessing large-scale patterns in data than for early detection or assessing spread.

### Challenge 2: Dealing with uneven spatial coverage

**Figure 4:**
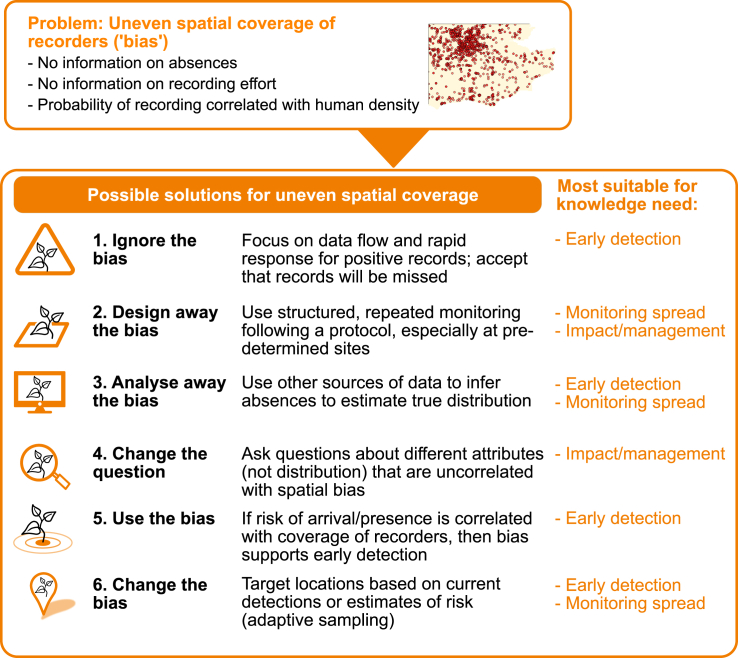
Summarizing the possible solutions for uneven spatial coverage of recorders as discussed in the main text We have indicated the main knowledge needs addressed by each proposed solution. The small map indicating uneven coverage of reports shows data from Pocock & Evans (2014) for London and south-east England.

The second major challenge regards the ability of the dataset to answer the questions of concern. Recording effort in IAS citizen science is inevitably unevenly distributed due to issues of accessibility, human population density, and recording preferences of volunteers.^71^^,^^136^^,^^137^ The greatest gap is that recording effort is often not known (especially for unstructured citizen science). Although this challenge is not unique to IAS citizen science, it does have important implications for different stages of the biological invasion process, especially in the establishment and spread phases (Figure 1). This is also relevant for later stages in the invasion process, when uneven coverage could lead to unbalanced experimental data, e.g., insufficient data from non-treatment sites. All this can result in citizen science data being insufficient for their intended purpose. This either leads to wasted volunteer contributions—because the data cannot be used—or leads to the risk of misleading conclusions—because analysis can lead to biased results.^71^^,^^138^ Here we review six opportunities to overcome the challenge from uneven coverage (Figure 4).

#### Ignore the bias

Ignoring the bias might seem initially unacceptable, but it is a practical and common approach for many IAS citizen science projects, especially those involved with early detection. Where members of the public are encouraged to submit observations of an IAS, it is difficult to know the true distribution of the “standing army” of potential recorders because it depends on so many different factors, such as public awareness and motivation to record, as well as spatial distribution of people (Figure 1). Yet, there is an asymmetry in data information^72^: a lack of reports may be due to the absence of the IAS or a lack of observers, but once an early detection sighting has been confirmed, action can take place. If the bias is ignored, it is important for project organizers to acknowledge this in their reporting.

#### Design away the bias

By using structured approaches with repeated sampling at sites, the challenge of uneven spatial coverage can be addressed (at least partially) through project design. Sampling locations can be selected in advance as a representative or random sample of the environment; from this it is possible to use weights to make inference about the whole population, e.g., about occupancy within a certain region.^139^ Following a protocol means that data on presence and absence (strictly speaking, “absence” is actually “non-detection”) of IAS can be obtained, thereby overcoming the challenge of using presence-only data, while also providing more consistent data on abundance.^126^

#### Analyze away the bias

Species distribution modeling can be used to account for uneven spatial coverage by combining presence/absence data with covariate data (e.g., habitat, latitude, altitude, or proximity to human habitation), e.g.,de Groot et al.^71^ Absence data may be difficult to obtain from unstructured citizen science (because there is little motivation for recorders to submit non-detections). In multispecies recording non-detections can be inferred from the records of other species to undertake occupancy modeling,^140^ which can help to account for variation in recorder effort and recording behaviour.^161^^,^^162^ Measures such as the “list length” of species can be used as proxies of recording effort in analysis of IAS distribution.^141^^,^^161^^,^^162^ One unstructured citizen science project focused on IAS inferred absences by asking for records of a diversity of IAS;^71^ another encouraged people to report more common species alongside the target IAS to assess recorder coverage.^142^

#### Change the question

One other way of addressing the challenge of uneven spatial coverage is to change the question. Typically project organizers ask about presence of IAS, but instead they could ask a different question, e.g., focusing on one of the many other attributes of species, such as abundance, individual size, and so on.^143^ If these attributes are not correlated with the uneven spatial coverage of recorders, then the results will be unbiased. This is obviously not possible if the question of concern is mapping the distribution (because the answer inherently depends on the distribution of recording effort) but could be valuable to assess impact of IAS, IAS management, or biological research on the species.^22^ Examples include estimating changing sex ratios of invading crabs^97^ or biological predators of an invading leaf-mining moth.^50^

#### Use the bias: Creating targeted surveillance

Humans and their activities are important sources of introduction, dispersal, and spread for many IAS. For instance, *Dikerogammarus villosus* killer shrimp is associated with lakes that are heavily used by recreation^144^ and spread of invasive plants is linked to transportation routes.^145^^,^^146^ These features are, by their nature, also correlated with likely recording effort. This means that detection of IAS is most likely in the places where introductions are most likely to occur, thus creating a positive bias that enhances early detection.

#### Change the bias: Targeting recording

Typically, citizen science for IAS can be regarded as either completely unstructured (people record what they want and when they want) or structured (people record at set places). However, an alternative is to develop adaptive citizen science monitoring.^86^ In an experimental test, using species distribution models to help target the activity of recorders was found to be useful to maximize efficiency of citizen science for IAS.^147^ Targeting can also be according to risk.^148^ Mobile technology such as smartphone apps means that this adaptive approach has great potential to provide live updates or “nudges” for recorders.

## What does the future hold for IAS citizen science?

### Where do we go from here?

Our review of citizen science and IAS shows that citizen science approaches are valuable across all the stages of the biological invasion process, but that the potential of citizen science for IAS surveillance, management, and research has not been fully realized (Table 2). Although its potential for early detection, recording spread, and public engagement is well-regarded^3^ and well-established^10^ (Figure 1B), we show that citizen science could be used more to help assess impacts and evaluate management (Table 2). We also conclude that more structured citizen science activities (e.g., using a fixed sampling protocol) would complement the growth of unstructured activities (“record what you want, when you want”; Table 1). Projects often focus on recording IAS presence, but, for greater value, this should be expanded to recording ecological traits like abundance or interactions, impacts on environmental, health, and socio-economics, and the presence or success of management efforts by local communities.

We accept that the examples in our review are predominantly from the Global North, where there is a long tradition of, and relatively high investment in, citizen science. Arguably, the potential for IAS citizen science to make a difference is even greater in the Global South where there is a greater lack of data on IAS,^3^ and where citizen science would bring additional benefits for public engagement and partnership building.^149^

The question, though, is how we go about seeking to fulfill this potential, both supporting the use of citizen science right across the biological invasion process, and across the world? In the conclusion of this review, we consider how we should continue to build on best practice and how co-development with stakeholders is vital to fulfill this potential.

### Building on best practices when innovating in citizen science

As new citizen science projects are developed, practitioners should continue to follow good practice, both specifically for IAS citizen science^10^^,^^93^^,^^150^^,^^151^ and more generally for example for running citizen science,^152^ data management,^8^ ethical practice,^62^^,^^153^ and volunteer recruitment.^154^ This is codified in the European Citizen Science Association’s 10 Principles of Citizen Science.^155^

As we have highlighted in the review, technology will continue to evolve to support IAS citizen science. Tools to facilitate detection and identification, such as image recognition and DNA analysis, will transform what is possible, especially as sensors become miniaturized and diagnostics give more rapid results.^156^ But new technology can also support engagement in citizen science, including personalized feedback with artificial intelligence^157^ and gamification to “nudge’ recorders toward behavior that benefits data quality.^86^ Citizen science data are of limited value if they are not shared, so continuing best practice in data sharing is essential. Efficient data flow requires good data infrastructure but also requires good metadata and use of data standards to improve interoperability and the re-use of data.^59^ Investment in data flow may not seem as glamorous as DNA analysis or artificial intelligence, but it is a crucial component of the success of citizen science for IAS.

### Co-development is needed to expand the reach and sustainability of citizen science

Although our review has focused on the role of citizen science for data provision for IAS surveillance, management, and research (including the challenges explored in the section “Challenges with use of citizen science for IAS surveillance, management and research”), we have emphasized the need to put people and their motivations at the heart of developments in citizen science. Indeed, one study considering ethical challenges to citizen science concluded that co-development was the solution to a wide range of challenges.^62^ Similarly, as practitioners seek to grow IAS citizen science in the Global South, it is likely to reveal further challenges in terms of data ownership, ethics, data infrastructure limitations, access to technology, and taxonomic knowledge.^158^^,^^159^ These will need to be addressed in collaboration with local people and through co-design of projects, protocols, and tools.^158^^,^^160^

Co-development is valuable because it gives all the different actors (potential volunteers, project practitioners, and data users) mutual understanding of each other’s motivations and aims. The practice of “community-based monitoring’ (designing the activity as “together we can …” rather than “you should …”) shows how co-development can work and how it improves the impact and long-term sustainability of environmental monitoring.^44^ Co-development also means that citizen science is oriented toward action and decision-making,^21^ designed for the benefit of those taking part as well as scientific use of the data.

## Conclusion

Citizen science is a tremendous tool to support IAS surveillance, management, and research. Citizen science alone will not meet all our needs for IAS data, yet it has become an important tool for IAS monitoring and research, thus complementing the role of scientists and biosecurity professionals. As we have discussed throughout this review, citizen science is diverse. Across the diversity of citizen science, it demonstrably is making a difference in IAS monitoring and research. Given the imperative for action on IAS,^3^^,^^7^ continued investment both in existing activities and for innovative citizen science is essential for us to better meet the need for better IAS data across the biological invasion process for the benefit of science and participants, and ultimately for the benefit of society as a whole.
