# Supplementary material for: Citizen science is a vital partnership for invasive alien species management and research
Source: iScience. 2023 Dec 3;27(1):108623. doi: 10.1016/j.isci.2023.108623 (PMC10776933; doi:10.1016/j.isci.2023.108623)
Supplement: Document S1. Table S1 [file mmc1.pdf]

iScience, Volume *27*

Supplemental information

Citizen science is a vital partnership for invasive alien species management and research

Michael J.O. Pocock, Tim Adriaens, Sandro Bertolino, René Eschen, Franz Essl, Philip E. Hulme, Jonathan M. Jeschke, Helen E. Roy, Heliana Teixeira, and Maarten de Groot

Citizen science is a vital partnership for invasive alien species management and research

Pocock, M.J.O., Adriaens, T., Bertolino, S., Eschen, R., Essl, F., Hulme, P.E., Jeschke, J.M., Roy, H.E., Teixeira, H., de Groot, M.

Table S1. The distribution of European projects that meet IAS policy and management needs, based on classification of the projects surveyed by Price-Jones et al. (2022). Each project can contribute to multiple outcomes spanning knowledge needs and management.

Contribution to IAS knowledge needs or management (number of knowledge need from Fig. 1)	% of projects in Price-Jones et al. (2022)	Definition from Price-Jones et al. (2022)
Risk assessment & transportation pathways (1)	23%	Prevention
Early detection (2)	59%	Early detection AND/OR<br>Early warning
Mapping distribution (3)	96%	Species presence AND/OR<br>Species abundance AND/OR<br>Mapping of alien species AND/OR<br>Collecting lists of species
Monitoring & surveillance (4)	69%	Rate of spread AND/OR<br>Monitoring or surveillance AND/OR<br>Changes in abundance
Evaluating impacts (5)	20%	Evidence of impacts on biodiversity AND/OR<br>Measuring impacts
Evaluating effectiveness of management (6)	20%	Effectiveness of management
Biological research (n/a)	24%	Experimental data AND/OR<br>Testing scientific hypothesis
Engagement (n/a)	69%	Engagement AND/OR<br>Education AND/OR<br>Awareness raising
